# Simultaneous paralogue knockout using a CRISPR-concatemer in mouse small intestinal organoids

**DOI:** 10.1016/j.ydbio.2016.10.016

**Published:** 2016-12-15

**Authors:** Amanda Andersson-Rolf, Alessandra Merenda, Roxana C. Mustata, Taibo Li, Sabine Dietmann, Bon-Kyoung Koo

**Affiliations:** a Wellcome Trust - Medical Research Council Stem Cell Institute, University of Cambridge, Gleeson Building, Tennis Court Road, Cambridge CB2 1QR, UK; b Department of Genetics, University of Cambridge, Cambridge CB2 3EH, UK

## Abstract

Approaches based on genetic modification have been invaluable for investigating a wide array of biological processes, with gain- and loss-of-function approaches frequently used to investigate gene function. However, the presence of paralogues, and hence possible genetic compensation, for many genes necessitates the knockout (KO) of all paralogous genes in order to observe clear phenotypic change. CRISPR technology, the most recently described tool for gene editing, can generate KOs with unprecedented ease and speed and has been used in adult stem cell-derived organoids for single gene knockout, gene knock-in and gene correction. However, the simultaneous targeting of multiple genes in organoids by CRISPR technology has not previously been described. Here we describe a rapid, scalable and cost effective method for generating double knockouts in organoids. By concatemerizing multiple gRNA expression cassettes, we generated a ‘gRNA concatemer vector’. Our method allows the rapid assembly of annealed synthetic DNA oligos into the final vector in a single step. This approach facilitates simultaneous delivery of multiple gRNAs to allow up to 4 gene KO in one step, or potentially to increase the efficiency of gene knockout by providing multiple gRNAs targeting one gene. As a proof of concept, we knocked out negative regulators of the Wnt pathway in small intestinal organoids, thereby removing their growth dependence on the exogenous Wnt enhancer, R-spondin1.

## Highlights

• Cloning of multiple guide RNAs into one concatemer vector in one single step.
• Simultaneous paralogue knockout in small intestinal organoids.
• The concatemer retroviral vectors can be used for transient transfection or viral transduction.

## 1 Introduction

It was discovered almost 70 years ago that DNA is the genetic material (Steinman and Moberg, 1994), and from that time onwards molecular genetics has made and continues to make essential contributions to our biological understanding of health and disease. Today, gain-of-function and loss-of-function approaches are frequently used to investigate gene function. CRISPR technology represents the most recent advance in gene editing tools and has revolutionized the speed and ease of genetic manipulation in diverse organisms.

Using CRISPR technology, double-strand breaks (DSBs) can be generated by the Cas9 endonuclease following its targeting to the desired genomic site under the guidance of a single stranded ‘guide’ RNA molecule (gRNA). Cas9-induced DSBs then activate the cell's endogenous DNA repair machinery, resulting in genetic repair either via the error-prone Non-Homologous End-Joining pathway (NHEJ) or via high-fidelity Homologous Recombination (HR). The latter allows the precise introduction of a desired nucleotide sequence (Cho et al., 2013, Cong et al., 2013, Jinek et al., 2013, Mali et al., 2013).

To date, genome-wide genetic knockout (KO) screens in flies, zebrafish, mice and human cells have been performed (Bassett et al., 2015, Cheloufi et al., 2015, Koike-Yusa et al., 2014, Leeb et al., 2014, Mali et al., 2013, Shah et al., 2015). These screens, although comprehensive, are based on the use of one or more gRNAs per gene, and the transduction of one cell with one single gRNA. This type of screen can be used to identify novel gene functions, but relies on phenotypic change being triggered by the deletion of a single gene. In the mammalian genome, a large number of genes have paralogues that are generated via gene duplication event(s) with potentially similar function (Jensen, 2001, Nehrt et al., 2011). Thus, single gene KO may not result in a clear phenotype due to the existence of functionally similar paralogue(s), a phenomenon known as genetic compensation. In order to negate such compensation it is necessary to knockout the gene of interest as well as its paralogue(s) to observe a clear phenotypic change. On a small scale this can be achieved by consecutive or simultaneous delivery of multiple gRNA vectors, but for larger scale approaches both the vector cloning and delivery has to be optimized.

To circumvent this problem, gRNA cassette multiplexing into one vector has been developed by many groups for mammalian systems (Albers et al., 2015; Kabadi et al., 2014; Maddalo et al., 2014; Sakuma et al., 2014; Vad-Nielsen et al., 2016; Wyvekens et al., 2014) as well as Drosophila (Port et al., 2014), *E. coli* (Cress et al., 2015), plants (Xie et al., 2015; Ma et al., 2015; Xing et al., 2014) and zebrafish (Yin et al., 2015). Golden gate cloning, based on the combination of Type II restriction enzymes and DNA ligase, is a popular cloning strategy used to multiplex gRNAs. Type II restriction enzymes can generate non-palindromic overhangs by cutting outside of their recognition sequence: this permits the assembly of multiple DNA fragments each with different overhang sequences (Engler et al., 2009). In this method, however, gRNAs are first cloned into individual vectors which are later combined into one final vector, thus requiring two cloning steps.

Here we report a cloning method allowing the assembly of multiple gRNAs in one single step, thus eliminating the need for additional cloning steps. Using small intestinal organoids, we generated double-mutant organoids with constitutively active Wnt signaling by knocking out all of the paralogues of key negative regulators in the Wnt signaling pathway.

## 2 Materials and methods

### 2.1 Cloning of the concatemer vector

#### 2.1.1 Cloning of backbone vectors with Bbs1 sites

The empty gRNA vector (41824, Addgene) was obtained from Addgene and used as a template for inverse PCR (Phusion DNA polymerase, M0530S, NEB) in order to incorporate two *Bbs*I cloning sites into the vector. For the inverse PCR, two primers were used: one primer (TTTTAGAGCTAGAAATAGCAAGTTAAAATAAGG) going in the forward direction was phosphorylated at the 5′ end while the other primer (Cassette 1: CGTCTTCTCGAAGACCCGGTGTTTCGTCCTTTCCACAAGAT, Cassette 2: CTGTCTTCTCGAAGACTCCGGTGTTTCGTCCTTTCCACAAGAT, Cassette 3: CTTGTCTTCTCGAAGACTTCCGGTGTTTCGTCCTTTCCACAAGAT, and Cassette 4: GTCTTCTCGAAGACCGGTGTTTCGTCCTTTCCACAAGAT) carried two inversely located *Bbs*I sites. Subsequent *Dpn*I treatment removed remaining methylated template vector before ligation and transformation into 10 G competent bacterial cells (60108-1, Cambridge Bioscience). The modified gRNA expression vectors containing *Bbs*I sites were transformed and isolated using a Qiagen spin miniprep kit (27106, Qiagen). These vectors were used as templates for the PCR amplification of each ‘cassette’ unit. The forward primer (AGATCTCCAAGGTCGGGCAGGAAGAGG) contains a *Bgl*II site in the overhang, binds to the upstream region of the U6 promoter and is common for all cassette units while the reverse primer (Cassette 1: GTCGACGAATTCGGATCCTTGTCATCGTCGTCCTTGTAGTCAAAAAAGCACCGACTCGGTGCCAC, Cassette 2: GTCGACGAATTCGGATCCGGCGTAGTCGGGCACGTCGTAGGGGTAAAAAAGCACCGACTCGGTGCCAC, Cassette 3: GTCGACGAATTCGGATCCCAGGTCCTCCTCTGAGATCAGCTTCTGCATTGATGCCATAAAAAAGCACCGACTCGGTGCCAC, and Cassette 4: GTCGACGAATTCGGATCCAAAAAAGCACCGACTCGGTGCCAC) binds to the 3′ end. Each reverse primer has a unique overhang, providing both a primer binding site for Sanger sequencing and recognition sites for the three restriction enzymes – *Bam*HI, *Eco*RI and *Sal*I. Each PCR-amplified cassette unit was cloned into the Pjet1.2 vector (ThermoFisher Scientific, K131). Using the vector containing the first cassette, the other three cassette units were added sequentially by digesting the destination vector with *Bam*HI (R0136, NEB) and *Sal*I (R3138, NEB) and the cassette to be inserted with *Bgl*II (R0144, NEB)and *Sal*I. This generated four different vectors containing; ‘Cassette 1’, ‘Cassette 1–2’, ‘Cassette 1–3’ and ‘Cassette 1–4’. The assembled cassette units were subsequently moved to the retroviral expression vector MSCV-puro (631461, Clontech) by restriction digestion using *Bgl*II and *Eco*RI (R0101, NEB).

#### 2.1.2 Multiple gRNA cloning

gRNAs were ordered as oligonucleotides (oligos) from Sigma Aldrich. Each oligo was composed of the 5′ and 3′ overhangs specific for the cassette into which the gRNA would be inserted as well as the gRNA sequence of choice (Table 1 and Supplementary Table 1). gRNAs with a low number of potential off-target sites were identified using the bioinformatics tool developed by the Zhang lab (http://crispr.mit.edu/). For each gRNA, two oligos corresponding to the sense and antisense sequence were ordered. Oligos were then mixed, phosphorylated and annealed in a single reaction using 10 µm of each oligo, T4 DNA ligase, T4 PNK and water in a 20 µl reaction with the following thermocycler settings; 37 °C for 30 min, 95 °C for 5 min, ramp down to 25 °C at 0.3 °C/min and 4 °C.

Golden gate cloning for 1 and 2 gRNAs was performed as previously described (Ran et al., 2013). For 3 and 4 gRNAs, the annealed and phosphorylated oligos were diluted 1:100 prior to Golden gate cloning using the following thermocycler settings; first 37 °C for 5 min and 21 °C for 5 min for 50 cycles, then 37 °C for 5 min and 4 °C. Treatment with Plasmid-safe (E3101K, Cambio) is optional but highly recommended to avoid false positive clones. The reaction mix was then transformed into 10 G bacteria (60108-1, Cambridge Bioscience) and candidate plasmids were isolated using a Qiagen midi prep kit (12145, Qiagen). Correctly assembled gRNA concatemers were identified by screening using *Bgl*II and *Eco*RI digestion and subsequent confirmation by Sanger sequencing.

### 2.2 Transfection of small intestinal organoids

Organoid transfection has previously been described (Schwank et al., 2013). In brief, prior to transfection organoids were cultured in Wnt3a-containing media supplemented with nicotinamide until they showed a cystic morphology. For transient transfection, 3–4 high density wells of a 48-well plate were collected into an Eppendorf tube and mechanically disrupted. Organoids were dissociated into single cells by treatment with TrypLE Express (12605010, Invitrogen), spun down and resuspended in 100 µl media without antibiotics (Advanced DMEM/F12 supplemented with 10 mM Hepes, Glutamax, 1× N2, 1× B27 (all from Invitrogen), and 1 µm N-acetylcysteine (Sigma) and containing the growth factors and inhibitors: 50 ng/ml EGF, 100 ng/ml noggin, CHIR99021 and Y-27632). For the transfection, 4 µl of Lipofectamine 2000 (11668019, Invitrogen) and a total amount of 1.6 µg of DNA (Cas9:concatemer in a 1:1 ratio) were added to separate tubes containing 50 µl Opti-MEM I Reduced Serum Medium (31985062, Gibco) and incubated separately for 5 min at room temperature before being pooled and incubated together for a further 25 min. Following addition of the Lipofectamine-DNA mixture to the single cell solution in one well of a 48-well plate, the plate was spun at 600×*g* for 1 h at 32 °C and then incubated at 37 °C in a tissue culture incubator for 6 h. Finally, the DNA-cell mixture was collected and spun down at 500×*g* for 5 min. The supernatant was removed and the cell pellet was resuspended in Matrigel (356231, Corning) and seeded into one well of a 48-well plate. Transfected organoids were cultured for 2–3 days before selection was started. Selection was performed in the organoid culture media without R-spondin1.

### 2.3 Small intestinal organoid culture

#### 2.3.1 Culture media

Murine small intestinal organoids were isolated and cultured as previously described (Sato et al., 2009).

#### 2.3.2 Picking of small intestinal organoids

Matrigel was gently disrupted by pipetting and transferred together with the media to a 35 mm dish (121 V, Thermofisher Scientific). Single organoids were picked under a light microscope, collected in Eppendorf tubes and then mechanically disrupted and seeded in Matrigel in a single well of a 48-well plate (CLS3548-100EA, Qiagen). Selection media was overlaid in the well once the Matrigel had solidified.

### 2.4 Sequencing

For sequencing, one single well of a 48-well plate of organoids derived from a single organoid clone was collected and mechanically disrupted. The organoid fragments were spun down at 600×*g* for 5 min at room temperature. The supernatant was removed and the pellet was resuspended in DirectPCR Lysis Reagent Ear (402-E, Viagen Biotech) and incubated at 60 °C for 3–5 h.

The DNA-lysis mix was diluted 1:10 and used as template for PCR amplification using Phusion DNA polymerase of the area surrounding the gRNA target region. The PCR product was cloned into Pjet1.2 and transformed into 10G competent bacteria (60108-1, Cambridge Bioscience). A minimum of 5 colonies were picked for each organoid clone and plasmid DNA was isolated from each of these colonies using a Qiagen spin miniprep kit (27106, Qiagen) to allow Sanger sequencing.

## 3 Results

### 3.1 Generation of a single ‘concatemer’ vector for multiple gRNA expression

Paralogues are genes within the same genome related by duplication (Jensen, 2001) and their presence makes it desirable to knockout multiple genes simultaneously in order to negate redundancy which may otherwise mask corresponding phenotypic changes by genetic compensation. Simultaneous knockout of several genes using CRISPR technology requires the efficient delivery of multiple gRNAs and Cas9. On a small scale this can be achieved by co-transfection of multiple gRNA plasmids or in vitro transcribed mRNAs. However, for a large scale screen and systems with low transfection efficiency it is desirable to have a single vector containing multiple gRNA cassettes.

To generate a single vector which allows the simultaneous expression of multiple gRNAs, the gRNA expression cassette with two inversely located *Bbs*I sites at the target sequence cloning site was concatemerized and cloned into the MSCV retroviral vector as described in ‘Section 2’ (2.1 Cloning of the concatemer vector). As a result, the four gRNA expression cassettes were combined into single MSCV vectors containing either one (‘Cassette 1’), two (‘Cassette 1–2’), three (‘Cassette 1–3’) or four (‘Cassette 1–4’) cassettes (Supplementary Fig. 1A).

**Fig. 1:**
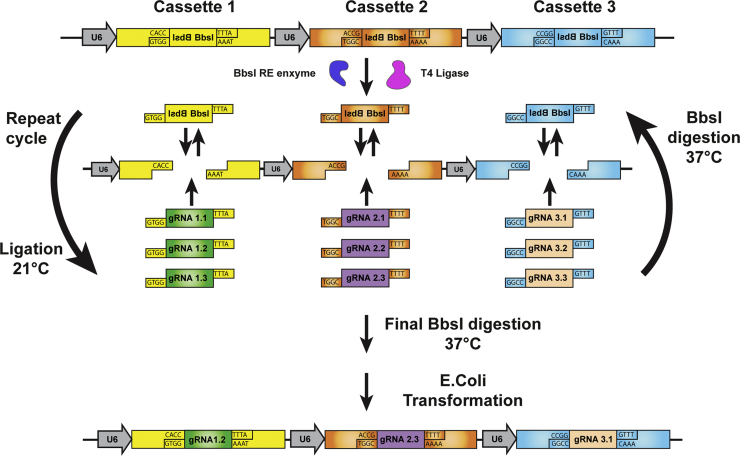
**Schematic image illustrating the Golden gate cloning of multiple gRNAs into the concatemer vector containing 3 gRNA expression cassettes**. Annealed oligos (gRNA targets), DNA ligase and the *Bbs*I restriction enzyme are mixed with the gRNA concatemer vector in a single reaction. Repeated temperature cycles facilitate repeated digestion (21 °C) and ligation (37 °C). *Bbs*I digestion generates the custom-designed overhangs unique for each cassette. During the ligation, gRNAs are integrated into the vector by cassette-specific integration, determined by the matching overhangs of the gRNA and the vector. If the original fragment containing the two *Bbs*I restriction sites is ligated back into the vector it will again be removed in the following round of digestion. In contrast, upon ligation of a gRNA the *Bbs*I restriction site is disrupted and hence the gRNA cannot be removed during the following rounds of digestion. Blue – *Bbs*I enzyme, pink – T7 DNA ligase, U6 – U6 promoter, gRNA1.1–1.3 represent different gRNAs for the same gene (e.g. gRNA1.1 – gRNA1 for gene 1).

Simultaneous cloning of multiple gRNAs into the concatemer vector is based on the unique, custom-designed overhangs of each individual gRNA expression cassette. gRNA oligos are ordered with overhangs matching that of the target cassette and can be annealed in a single reaction or multiple individual reactions (Table 1). Annealed oligos are cloned into the concatemer vector by Golden gate assembly, where continuous cycles of digestion and ligation facilitate incorporation of the gRNAs into the correct cassette, so resulting in the final vector (Fig. 1).

Cloning of gRNAs into the ‘Cassette 1’ and ’Cassette 1–2’ concatemer vectors worked efficiently with previously described protocols (Ran et al., 2013) but for the efficient simultaneous insertion of 3 or 4 gRNAs, conditions were optimized to include a vector:insert ratio of 1:5 and an increased number of cycles for Golden gate assembly. Using this optimized protocol, multiple gRNAs were cloned into the ‘Cassette 1–3’ or ‘Cassette 1–4’ with at least 25% efficiency (Supplementary Table 1). Successful gRNA integration was confirmed by restriction digestion. When performing restriction digestion with *Bgl*II and *Eco*RI, expected band sizes correspond to the number of expression cassettes, with the size of a single cassette being ~400 bp (Supplementary Fig. 1A,B). Additional restriction using *Bbs*I allows detection of false positives, as successful gRNA insertion destroys the *Bbs*I site of the expression cassette, thus inhibiting digestion (Supplementary Fig. 1B).

### 3.2 Simultaneous knockout of functionally related paralogues in small intestinal organoids with the concatemer vector

To validate the use of the concatemer vector we chose primary mouse small intestinal organoid culture. Although this 3D culture system is rapidly becoming popular, as it closely resembles the in vivo tissue, it is still relatively difficult to transfect mouse intestinal organoids using simple lipofection methods when compared to HEK293 and mouse embryonic stem cells. Here, we aimed to knock out paralogues acting as negative regulators of the Wnt signaling pathway in organoid culture using our concatemer vector system.

The Wnt pathway plays an essential role in the maintenance of small intestinal stem cells (ISCs) in vitro as well as in vivo (Gregorieff and Clevers, 2005, Sato et al., 2009). Upon the binding of Wnt to its receptor Frizzled and co-receptor Lrp5/6 (lipoprotein receptor related protein 5/6), the activated receptor recruits Dvl (Dishevelled), which sequesters the destruction complex at the plasma membrane thus inactivating it. This results in an increased level of β-catenin, which translocates to the nucleus where it interacts with the Tcf/Lef transcription factors and induces the transcription of Wnt target genes. Rnf43 and Znrf3 form another axis of regulation in the Wnt pathway. These negative feedback regulators are expressed upon Wnt stimulation and prevent prolonged Wnt activation by inhibiting the Frizzled and Lrp5/6 complex. Upon R-spondin stimulation, Rnf43/Znrf3 are recruited to the Lgr4/5/6 and R-spondin complex and hence abrogate this negative feedback loop, resulting in enhanced Wnt activation.

**Fig. 2:**
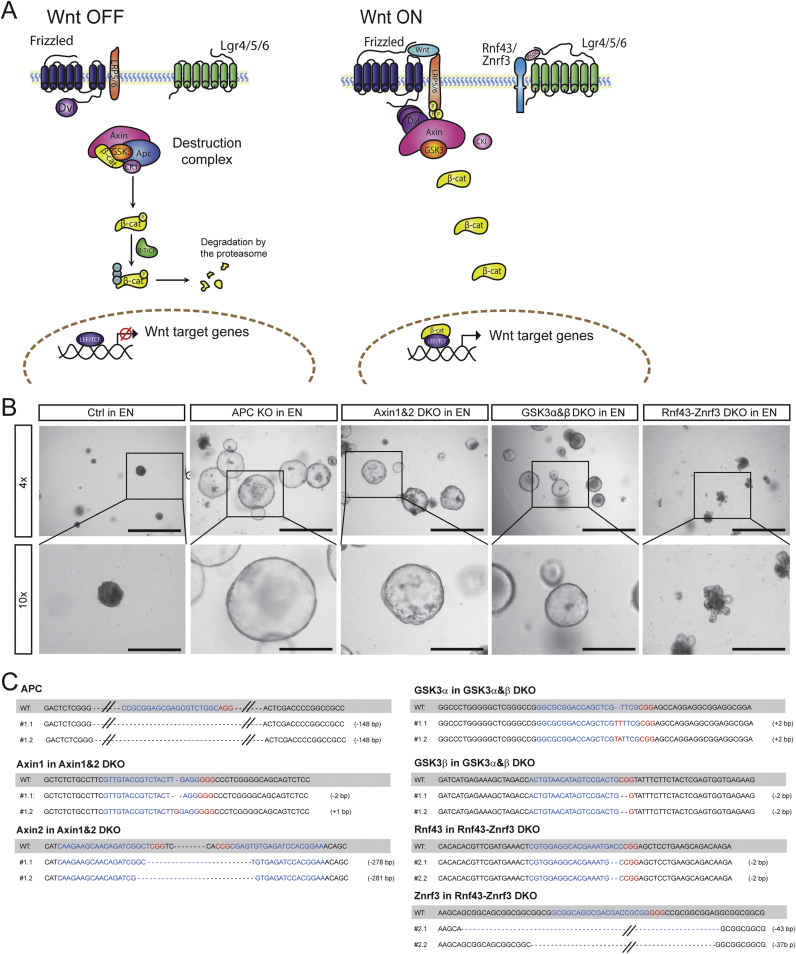
**Simultaneous paralogue knockout in small intestinal organoids using gRNA concatemer vectors.** (A) Schematic image of the Wnt signaling pathway. In the absence of Wnt, the destruction complex consisting of Apc (Adenomatous polyposis coli) and Axin, which act as scaffolds, together with the two kinases GSK3 (Glycogen synthase kinase 3) and CK1 (Casein Kinase 1) mediates the degradation of β-catenin, resulting in inhibition of Wnt signaling. For more details on the Wnt pathway the reader is advised to refer to the main text. (B) Representative images of small intestinal organoids in which negative regulators of the Wnt pathway have been knocked out, thus rendering their growth independent of addition of the Wnt enhancer R-spondin1. DKO – double knockout, EN- Egf- and Noggin-containing medium lacking R-spondin1. 4× scale bar 1000 µm. 10× scale bar 400 µm. (C) Sequence data confirm that organoids growing in the absence of R-spondin1 contain mutations in Wnt pathway regulators. The Apc knockout has an 148 bp deletion in both alleles. The Axin1&2 double knockout has a deletion of 1 and 2 bp in the alleles of Axin1 and a 278 and 281 bp deletion in the alleles of Axin2. The GSK3 double knockout has a 2 bp insertion in both alleles of GSK3α and a 2 bp deletion in both alleles of GSK3β. The Rnf43-Znrf3 double knockout has a 2 bp deletion in both alleles of Rnf43 and a 37 and 43 bp deletion in the alleles of Znrf3. Number next to sequences indicate clone number and allele.

*In vitro*, removal of the growth factor R-spondin1, a potent Wnt enhancer, results in rapid death of the cultures within 3–4 days. Only organoids subject to constitutively active Wnt signaling, for example following mutation of the Adenomatous polyposis coli (Apc) gene or treatment with the Glycogen Synthase Kinase 3 (GSK3) inhibitor (CHIR99021), can survive withdrawal of R-spondin1 (Sato et al., 2011, Schwitalla et al., 2013, Yin et al., 2014). Thus, small intestinal organoids represent a sensitive model system in which the simultaneous knockout of key Wnt pathway members and their paralogues can be easily tested by a clear phenotypic readout, namely survival following R-spondin1 withdrawal. Constitutive Wnt pathway activation can be achieved by inhibiting the destruction complex consisting of Apc, GSK3, Axin and CK1 (casein kinase 1) or by disrupting the negative feedback loop formed by the two E3 ubiquitin ligases Rnf43 and Znrf3 (RZ) (Fig. 2A).

Concatemer vectors were generated for the abovementioned proteins and their paralogues (GSK3α and GSK3β, Axin1 and Axin2, and RZ). Prior to transfection, organoids were cultured in Wnt3a-containing media until they adopted a cystic morphology, after which they were disrupted into fragments containing 1–5 cells and co-transfected with an equal amount of concatemer vector and Cas9 plasmid (Addgene, #41815) using Lipofectamine 2000. Importantly, the organoid-DNA mix was spun at 32 °C for 1 h to increase the transfection efficiency. Organoids were cultured in media containing Wnt3a and ROCK inhibitor for 3 days following transfection, after which selection was initiated by the removal of R-spondin1 from the culture media. Following 10 days of selection, surviving organoid clones were picked, expanded and genotyped by Sanger sequencing to confirm successful KO. The recovered organoid clones survived without R-spondin (Fig. 2B) and showed disruption of both paralogues for Axin1&2, GSKα&β and RZ as appropriate, confirming the functionality of the concatemer vector (Fig. 2C).

## 4 Discussion

CRISPR technology has made genome editing both simple and fast and has facilitated genome-wide knockout screens in mouse and human cells (Koike-Yusa et al., 2014, Leeb et al., 2014, Wang et al., 2013). Although powerful, the presence of paralogues might conceal potential phenotypes when knocking out only single genes. Therefore, it is desirable to not only knock out the gene of interest but also its paralogues. Here we present a method allowing the one step generation of a single concatemer vector simultaneously expressing multiple gRNAs. Based on Golden gate cloning, this rapid method requires only one cloning step and the generated vector can immediately be used for plasmid transfection or retroviral transduction. In addition, gRNAs can be easily ordered as oligos with customized overhangs. It is of note that the gRNA sequence should not contain any *Bbs*I recognition sequences, as this restriction enzyme is used during the vector assembly. The concatemer method is expected to be compatible with other cell lines and organoids as long as they can be maintained following single cell dissociation.

Using the concatemer vector, we demonstrated simultaneous disruption of genes of the Wnt pathway in the context of small intestinal organoids, thus allowing organoids to survive in the absence of the Wnt enhancer R-spondin1. Rnf43/Znrf3 double KO organoids display a budding morphology compared to Apc, Axin1/2, GSK3α/β which are round and cystic. This is caused by the Wnt dependency of Rnf43/Znrf3 double KO organoids, in which buds are formed around Wnt-secreting Paneth cells. On the other hand, the other mutants exhibit autonomous Wnt activation due to the lack of a functional destruction complex, which leads to uniform growth activity and round cystic organoids. Other potential uses for the concatemer vector include the cloning of multiple gRNAs targeting one gene into a single vector in order to increase efficiency for wildtype- and nickase-mediated gene editing, and the generation of a paralogue KO library. Moreover, the design of the concatemer vector allows the insertion of additional cassettes (A *Bgl*II-*Sal*I concatemer fragment to *Bam*HI and *Sal*I sites of another concatemer vector, please note that *Bgl*II and *Bam*HI are compatible and their ligation destroys the restriction site) to increase the number of gRNAs in one concatemer vector. Another alternative to increase the targeting numbers would be to simply co-transfect multiple concatermers. In summary our approach is a rapid and efficient alternative for the cloning of multiple gRNAs to allow simultaneous knock out of multiple genes, here demonstrated using the organoid system.

## Author contributions

A.A-R. and B-K.K. wrote the manuscript. A.A-R. and A.M. performed the experiments. A.A-R. generated the concatemer vectors, optimized the cloning efficiency and performed knockout of the Wnt paralogues in small intestinal organoids. A.M. cloned additional genes into the concatemer vector. R.M. helped with preparing Wnt3a conditioned media and growth factors. S. D. helped with gRNA selection and T.L. helped with the optimization of the gRNA cloning efficiency. B-K.K. supervised the project.

## Competing financial interest

The authors declare no competing financial interest.

## Figures and Tables

**Table 1:** Sequence of the customized overhangs for cloning into the different cassettes.

	Cassette 1	Cassette 2	Cassette 3	Cassette 4
Sequence 5′ to 3′	CACCGG[target]GT	ACCGG[target]G	CCGG[target]	ACACCGG[target] GTT
Sequence 5′ to 3′	TAAAAC[rc-target]CC	AAAAC[rc-target]C	A AAC[rc-target]	CTAAAAC[rc-target]CCG

## Appendix A Supplementary material

Supplementary material Supplementary Fig. 1. Representative gel images of restriction digestion patterns following cloning of gRNAs into the concatemer vector. (A) Representative image of the sizes of the ‘Cassette 1’ to ‘Cassette 1–4’ vectors following digestion with *Bgl*II and *Eco*RI. (B) Pattern obtained following successful cloning of gRNA into the ‘Cassette 1–3’ concatemer (lanes 1–3) and ‘Cassette 1–4’ concatemer (lanes 4–7) following digestion with *Bgl*II and *Eco*RI (top) and *Bbs*I (bottom). *Bgl*II and *Eco*RI digestion confirms the size of the insert. *Bbs*I digestion shows no valid *Bbs*I site in the plasmid, confirming the successful cloning of gRNAs. This is because the insertion of the gRNA fragments disrupts the *Bbs*I sites.

.

Supplementary material Supplementary Table 1. Primer sequences for gRNAs cloned into the concatemer vector targeting the listed genes and paralogues.
