# Supplementary figures and images for: Simultaneous paralogue knockout using a CRISPR-concatemer in mouse small intestinal organoids
Source: Dev Biol. 2016 Dec 15;420(2):271–7. doi: 10.1016/j.ydbio.2016.10.016 (PMC5161140; doi:10.1016/j.ydbio.2016.10.016)

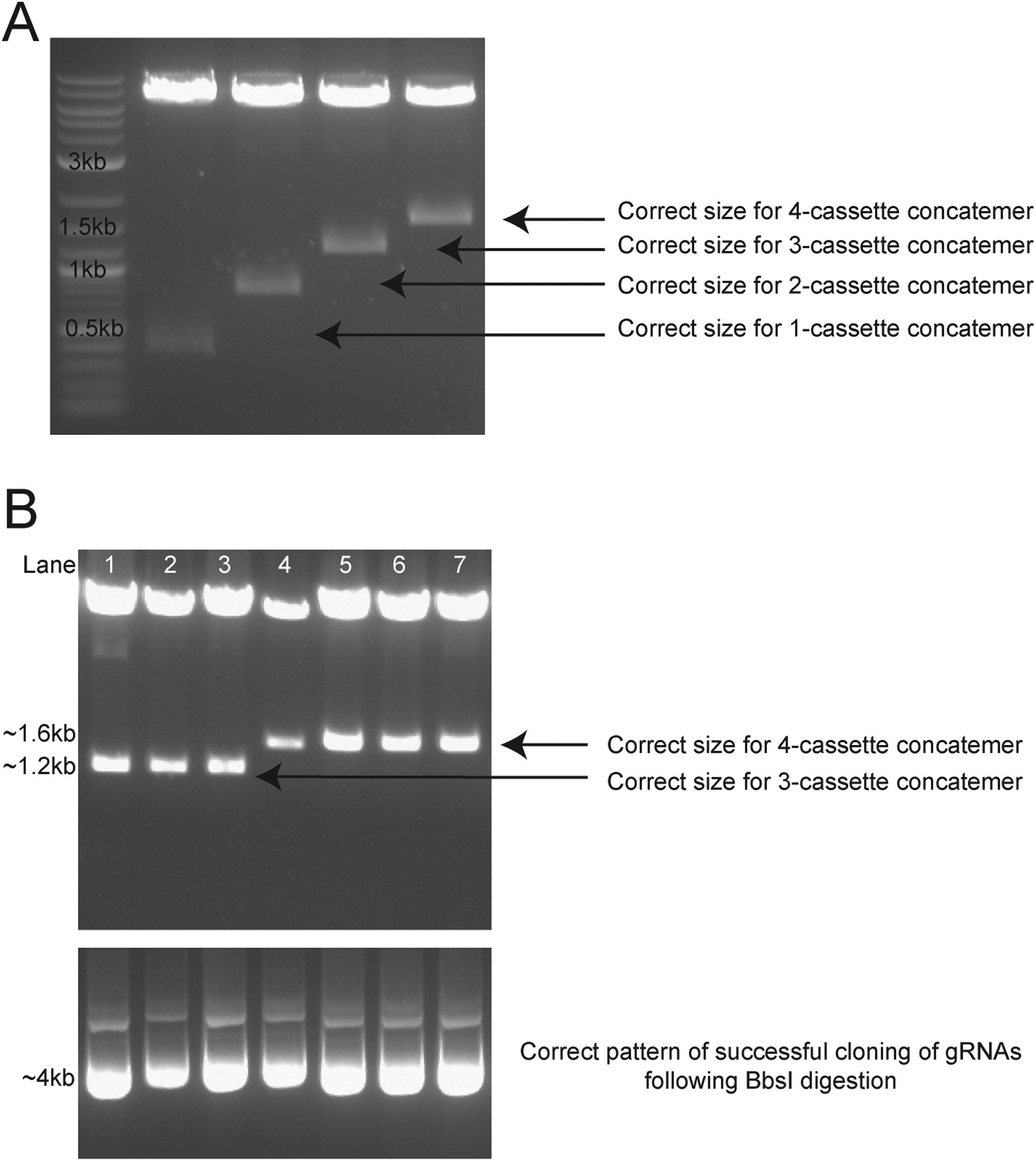

Supplement: Supplementary file 1 — Supplementary material Supplementary Fig. 1. Representative gel images of restriction digestion patterns following cloning of gRNAs into the concatemer vector. (A) Representative image of the sizes of the ‘Cassette 1’ to ‘Cassette 1–4’ vectors following digestion with BglII and EcoRI. (B) Pattern obtained following successful cloning of gRNA into the ‘Cassette 1–3’ concatemer (lanes 1–3) and ‘Cassette 1–4’ concatemer (lanes 4–7) following digestion with BglII and EcoRI (top) and BbsI (bottom). BglII and EcoRI digestion confirms the size of the insert. BbsI digestion shows no valid BbsI site in the plasmid, confirming the successful cloning of gRNAs. This is because the insertion of the gRNA fragments disrupts the BbsI sites. [file mmc1.zip › mmc1.tif]

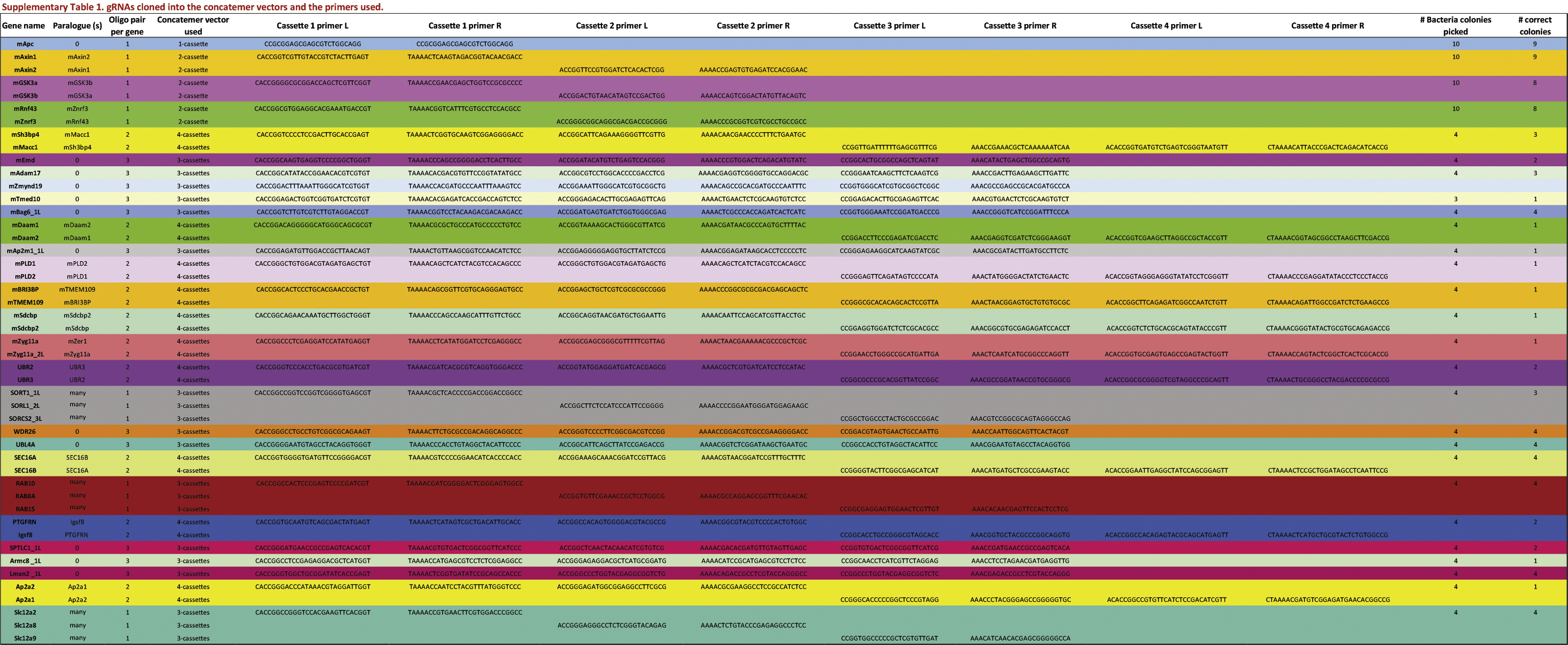

Supplement: Supplementary file 2 — Supplementary material Supplementary Table 1. Primer sequences for gRNAs cloned into the concatemer vector targeting the listed genes and paralogues. [file mmc2.zip › mmc2.tif]
